# Supplementary material for: Gene Expression of Putative Pathogenicity-Related Genes in Verticillium dahliae in Response to Elicitation with Potato Extracts and during Infection Using Quantitative Real-Time PCR
Source: Pathogens. 2021 Apr 23;10(5):510. doi: 10.3390/pathogens10050510 (PMC8146963; doi:10.3390/pathogens10050510)
Supplement: Supplementary file 1 [file pathogens-10-00510-s001.zip › Supplementary Table S-1.pdf]

Supplementary Table S1 Signal peptides prediction by SignalP 5.0

Protein ID	Prediction	Likelihood of SP(Sec/SPI)	Likelihood of Other	CS Position
Thioredoxin (VdTRX) (VDAG_04529)	Other	0.000779	0.999221	
NADH-ubiquinone oxidoreductase (VDAG_09026)	Other	0.001626	0.998374	
Pyruvate dehydrogenase E1 component subunit beta (VdPDHB) (VDAG_01642)	Other	0.000773	0.999227	
Ubiquitin-conjugating enzyme variant MMS2 (VDAG_05365)	Other	0.001165	0.998835	
HAD-superfamily hydrolase (VDAG_08490)	Other	0.000885	0.999115	
Serine 3-dehydrogenase (VDAG_09532)	Other	0.049198	0.950802	
Wos2 (VDAG_08865)	Other	0.001621	0.998379	
Ras-GAP like protein (VDAG_01012)	Other	0.000241	0.999759	
Xanthine dehydrogenase (VDAG_07735)	Other	0.002117	0.997883	
Myo-inositol 2-dehydrogenase (VDAG_08205)	Other	0.00212	0.99788	
DNA-(apurinic or apyrimidinic site) lyase (DNA AP lyase) (VDAG_02445)	Other	0.000489	0.999511	
Serine/threonine-protein kinase (VDAG_04632)	Other	0.004421	0.995579	
Glucan endo-1,3-alpha-glucosidase agn1 (VDAG_04101)	SP(Sec/SPI)	0.986551	0.013449	CS pos: 22-23 (between amino acid "VQA" and "KS"); Probability: 0.9129
DNA repair protein RAD51 (VDAG_08796)	Other	0.000732	0.999268	
Nuc-1 negative regulatory protein preg (PREG) (VDAG_06766)	Other	0.000642	0.999358	

Note:

Protein sequences were obtained from Verticillium comparative genomic project of the Broad Institute (https://www.broadinstitute.org/scientific-community/science/ projects/fungal-genome-initiative/verticillium-comparative-genomics-pro), and input into SignalP 5.0 [25] for signal peptides prediciton (http://www.cbs.dtu.dk/services/SignalP/);

SP(Sec/SPI): Secretory signal peptides transported by the Sec (Sec: secretory) translocon and cleaved by Signal Peptidase I;
Other: Protein do not possess a secretory singnal peptide;
CS position (CS pos): Cleavage site position for Signal Peptidase.
