# Supplementary material for: Gene Expression of Putative Pathogenicity-Related Genes in Verticillium dahliae in Response to Elicitation with Potato Extracts and during Infection Using Quantitative Real-Time PCR
Source: Pathogens. 2021 Apr 23;10(5):510. doi: 10.3390/pathogens10050510 (PMC8146963; doi:10.3390/pathogens10050510)
Supplement: Supplementary file 1 [file pathogens-10-00510-s001.zip › Supplementary Table S-2.pdf]

Supplementary Table S2 Effector prediction by Effector 1.0 & 2.0

Protein ID	Prediction by EffectorP 1.0	Probability (EffectorP 1.0)	Prediction by EffectorP 2.0	Probability (EffectorP 2.0)
Thioredoxin (VdTRX) (VDAG_04529)	Non-effector	1	Non-effector	0.966
NADH-ubiquinone oxidoreductase (VDAG_09026)	Non-effector	0.994	Non-effector	0.945
Pyruvate dehydrogenase E1 component subunit beta (VdPDHB) (VDAG_01642)	Non-effector	1	Non-effector	0.722
Ubiquitin-conjugating enzyme variant MMS2 (VDAG_05365)	Effector	0.946	Effector	0.72
HAD-superfamily hydrolase (VDAG_08490)	Non-effector	0.663	Non-effector	0.667
Serine 3-dehydrogenase (VDAG_09532)	Effector	0.582	Non-effector	0.56
Wos2 (VDAG_08865)	Non-effector	1	Non-effector	0.912
Ras-GAP like protein (VDAG_01012)	Non-effector	1	Non-effector	0.991
Xanthine dehydrogenase (VDAG_07735)	Non-effector	1	Non-effector	0.956
Myo-inositol 2-dehydrogenase (VDAG_08205)	Non-effector	0.951	Effector	0.572
DNA-(apurinic or apyrimidinic site) lyase (DNA AP lyase) (VDAG_02445)	Non-effector	1	Non-effector	0.985
Serine/threonine-protein kinase (VDAG_04632)	Non-effector	1	Non-effector	0.988
Glucan endo-1,3-alpha-glucosidase agn1 (VDAG_04101)	Non-effector	1	Non-effector	0.99
DNA repair protein RAD51 (VDAG_08796)	Non-effector	0.999	Non-effector	0.798
Nuc-1 negative regulatory protein preg (PREG) (VDAG_06766)	Non-effector	1	Non-effector	0.911

Note:
Protein sequences were obtained from Verticillium comparative genomic project of the Broad Institute (https://www.broadinstitute.org/scientific-community/science/ projects/fungal-genome-initiative/verticillium-comparative-genomics-pro), and input into EffectorP 1.0 [27] & 2.0 [26] for effector prediciton (http://effectorp.csiro.au/)
