# Supplementary material for: Gene Expression of Putative Pathogenicity-Related Genes in Verticillium dahliae in Response to Elicitation with Potato Extracts and during Infection Using Quantitative Real-Time PCR
Source: Pathogens. 2021 Apr 23;10(5):510. doi: 10.3390/pathogens10050510 (PMC8146963; doi:10.3390/pathogens10050510)
Supplement: Supplementary file 1 [file Supplementary Figure S-1.pdf › Supplementary Figure S-1.pdf]

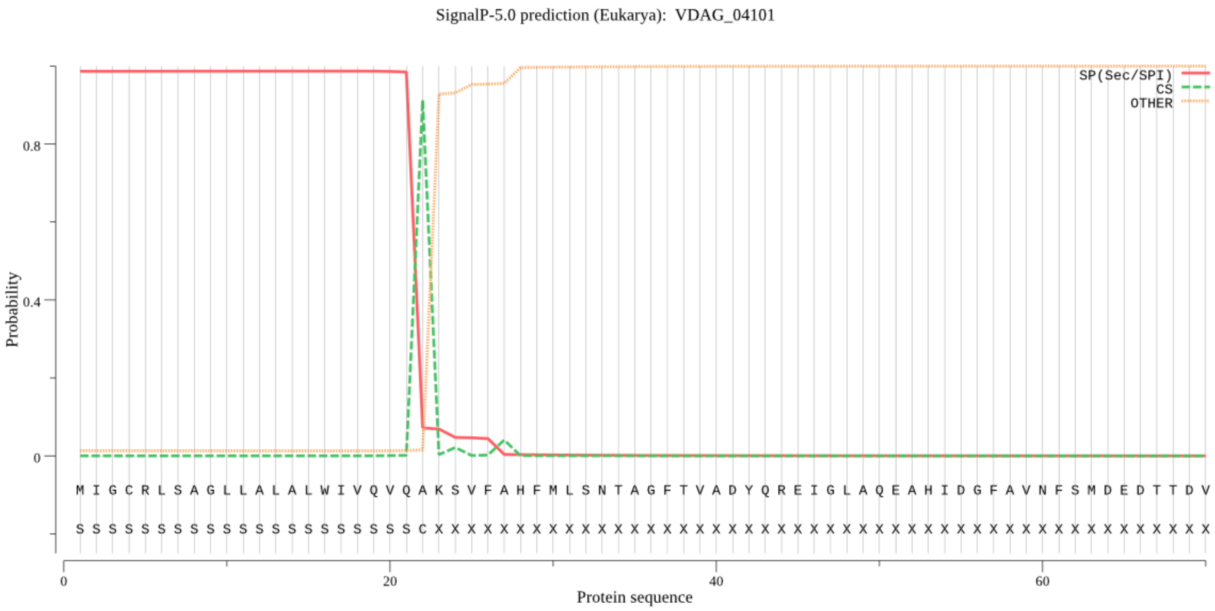

Supplementary Figure S1 Signal peptides prediction of Glucan en-do-1,3-alpha-glucosidase agn1 by SignalP 5.0

Protein sequences were obtained from Verticillium comparative genomic project of the Broad Institute (https://www.broadinstitute.org/scientific-community/science/ projects/fungal-genome-initiative/verticillium-comparative-genomics-pro), and input into SignalP 5.0 [25] for signal peptides prediciton (http://www.cbs.dtu.dk/services/SignalP/);

SP(Sec/SPI): Secretory signal peptides transported by the Sec (Sec: secretory) translocon and cleaved by Signal Peptidase I;

Other: Protein do not possess a secretory singnal peptide;

CS position (CS pos): Cleavage site position for Signal Peptidase;

Cleavage site position for Glucan en-do-1,3-alpha-glucosidase agn1 predicted by SignalP 5.0 is shown as No. 22-23 amino acid (between amino acid "VQA" and "KS") with a probability of 0.9129.
